# A SNP-Based Genome-Wide Association Study to Mine Genetic Loci Associated to Salinity Tolerance in Mungbean (*Vigna radiata* L.)

**DOI:** 10.3390/genes11070759

**Published:** 2020-07-07

**Authors:** Caleb Manamik Breria, Ching-Hsiang Hsieh, Tsair-Bor Yen, Jo-Yi Yen, Thomas J. Noble, Roland Schafleitner

**Affiliations:** 1 World Vegetable Center, Biotechnology and Molecular Breeding, Shanhua, Tainan 74199, Taiwan; joyce.yen@worldveg.org; 2 Department of Plant Industry, National Pingtung University of Science and Technology, Neipu, Pingtung 912, Taiwan; hsieh@mail.npust.edu.tw; 3 Department of Tropical Agriculture and International Cooperation, National Pingtung University of Science and Technology, Neipu, Pingtung 912, Taiwan; tbyen@mail.npust.edu.tw; 4 Centre of Tropical Agriculture and Biocommodities, Queensland University of Technology, Brisbane, Queensland 4000, Australia; t2.noble@qut.edu.au

**Keywords:** legume, salt-stress, genome-wide association mapping, mungbean, single nucleotide polymorphism, molecular markers

## Abstract

Mungbean (*Vigna radiata* (L.) R. Wilzeck var. *radiata*) is a protein-rich short-duration legume that fits well as a rotation crop into major cereal production systems of East and South-East Asia. Salinity stress in arid areas affects mungbean, being more of a glycophyte than cereals. A significant portion of the global arable land is either salt or sodium affected. Thus, studies to understand and improve salt-stress tolerance are imminent. Here, we conducted a genome-wide association study (GWAS) to mine genomic loci underlying salt-stress tolerance during seed germination of mungbean. The World Vegetable Center (WorldVeg) mungbean minicore collection representing the diversity of mungbean germplasm was utilized as the study panel and variation for salt stress tolerance was found in this germplasm collection. The germplasm panel was classed into two agro-climatic groups and showed significant differences in their germination abilities under salt stress. A total of 5288 SNP markers obtained through genotyping-by-sequencing (GBS) were used to mine alleles associated with salt stress tolerance. Associated SNPs were identified on chromosomes 7 and 9. The associated region at chromosome 7 (position 2,696,072 to 2,809,200 bp) contains the gene *Vradi07g01630*, which was annotated as the ammonium transport protein (AMT). The associated region in chromosome 9 (position 19,390,227 bp to 20,321,817 bp) contained the genes *Vradi09g09510* and *Vradi09g09600*, annotated as OsGrx_S16-glutaredoxin subgroup II and dnaJ domain proteins respectively. These proteins were reported to have functions related to salt-stress tolerance.

## 1. Introduction

A significant portion of the world’s land area is either salt- or sodium-affected. Soil salinity can be due to natural processes, or due to human intervention, such as extensive fertilization or wrong irrigation practices [1]. An environment is classified as being saline when the electrical conductivity exceeds 4 dSm^−1^ which amounts to a little over 40 mM NaCl [2].

Plants encounter osmotic imbalances and toxicity effects once the salt concentration is higher than the optimum concentration. Through time, salinity can undermine plant growth in two ways. High salt concentrations of the growing medium impede water absorption via the roots (osmotic effect); and high ionic concentrations (most common Na^+^, but also other ions) within plant tissues are toxic to the plant metabolism (toxicity effect) [3]. The osmotic stress phase begins immediately when the salt concentration outside the roots increases above the optimum level. Physiological effects include the reduction of shoot growth with slow emergence of new leaves and lateral buds [2]. The ionic phase of the salinity response begins when ions accumulate in plant tissues. Mature leaves having already reduced expansion rates show premature senescence and die [1]. When the loss of old leaves exceeds the production of new ones, the photosynthetic capacity of the plant is impeded, resulting in limited carbohydrate production, hence the growth of young developing leaves is stunted. 

Generally, salinity stress affects the whole life cycle of the plant and impedes germination, growth, development and reproduction [3]. At the germination stage, germination rate is reduced, compromising root development and plant establishment. The situation happens when high ion concentrations inhibit water uptake [4,5], subsequently leading to both ion toxicity and osmotic stress. Upon successful seed germination, the early salt response of seedlings can be observed in the form of increased root growth density (hypertrophy) to improve resistance and rapid acceleration of plant establishment [3]. However, this is not the sole response under salinity and different plants show varying ratios of root to shoot mass [6]. At later developmental stages, the excessive accumulation of ions in shoots disrupts photosynthetic activity resulting in premature leaf senesce [7]. Plant growth inhibition is often associated with a reduction in photosynthetic activity [8]. In most cases, it instigates programmed cell death (PCD). At the reproductive stage, salinity affects vital physiological functions inhibiting nutritional balance [9] and hormonal regulation [10]. This leads to increased rate of flower abortion, herewith reducing the number of flowers and seeds and eventually impeding yield [9]. 

Plant strategies to cope with elevated salt concentrations have evolved over time depending on species and the environment they thrive in [11]. These strategies can either be—(i) adaptive mechanisms (ability to survive the adverse condition) or (ii) specific mechanisms (habits to avoid the stress condition) [12]. Mechanisms of genetic control of salt tolerance are complex and generally implicate multiple genes and functions. Three distinct types of salt tolerance mechanisms were reported [3].

(1) Tolerance to osmotic stress—under osmotic stress (caused through salt or drought), changes in the turgor of leaves cause stomata closure, reducing transpiration and ensuring water retention. Loss in turgidity triggers changes in hormones and gene expression through upregulation of abscisic acid (ABA), leading to increased osmotic adjustment to minimize salinity effects by maintaining foliar turgidity [6]. Osmotic adjustment may help cells regain water and turgidity [13].
(2) Ion exclusion—ion (most often Na^+^) exclusion by plant roots prevents Na^+^ accumulation in leaves and avoids the toxic effect of higher ion concentrations, resulting in premature senescence and death of mature leaves and stunted growth of young leaves. Excluding excessive ions entails the up- and downregulation of ion channels and transporter genes to allow a tighter control of Na^+^ transport throughout the plant [3]. Ion exclusion occurs in some glycophytes and is very effective in halophytes. It happens right at the roots, preventing salts reaching the plant’s aerial parts. Development of saline vesicles at the leaf epidermis facilitates excretion of excessive salts to prevent accumulation in different plant organs. Part of this mechanism is due to salt deposition on mature leaves (leaf senescence), while avoiding accumulation in young developing leaves [6]. This mechanism avoids or postpones problems caused by Na^+^, but requires compensation through K^+^ uptake. Otherwise, there will be a greater need for organic solutes for osmotic adjustment. Again, synthesizing solutes jeopardizes the plant’s energy balance. Thus, the plant must be able to cope with both strategies of ion toxicity and turgor loss at the same time [3].
(3) Tissue tolerance to ions—tissue tolerance involves a tolerance mechanism of Na^+^, or other cations, and in some species it involves Cl^−^. It is achieved at cellular and intracellular levels where salt ions are compartmentalized. This mechanism is to avoid toxic concentration in cytoplasm and more specifically in the mesophyll cells of the leaves [3]. Tissue tolerance to ions allows for increased survival and retention of mature leaves. It is achieved through synthesis and accumulation of compatible solutes (CS) [14]. The CS are vital for plant osmotolerance and provide adaptive roles in osmotic adjustment during salt accumulation [15]. However, the functions of CS are not limited to osmotic adjustment. They (CS) can also act as low molecular chaperons. Their hydrophilic property gives them the ability to replace water on protein or membrane surfaces [14,16] and herewith protect enzymes from being denatured and stabilize membranes. They also mitigate the effect of reactive oxygen species (ROS) on cell functions [16]. Compatible solutes, being small water-soluble molecules, when present in high concentrations, remain neutral with respect to cellular functions [17]. They consist of either nitrogen-rich compounds like amino acids, betaines and amines or sugars, polyols and organic acids [14,18].

Responses to salt stress in leguminous crops have been discussed [19,20,21,22,23,24,25]. It was found that NaCl when added at increasing concentrations to germinating seeds of black gram (*Vigna mungo* L.) caused a decline in germination rate, root and shoot length and fresh biomass. The accumulation of proline then contributes to decreasing enzyme activity of catalase, peroxidase and polyphenol oxidase [19]. Boron and calcium, when added to germinating pea (*Pisum sativum*) seeds, were found to reduce the NaCl-mediated inhibition of seed germination and seedling growth [20]. A study on the interactions between seed size and NaCl on germination of chickpea (*Cicer arietium* L.) concluded that the bigger the seed size, the lower the germination index and root and shoot length, with prolonged mean germination time [21]. In Mediterranean legume pastures, five self-regenerating pastures (*Medicago polymorpha*, *Melilotus siculus*, *Trifolium subterraneum*, *T. michelianum* and *T. tomentosum*) were assessed for their tolerance mechanism in germination under NaCl stress. Results showed the annual seed coat impermeability protects the developing seed against the toxic effect of salinity during germination [22]. In faba bean (*Vicia faba*), one of the most salt-tolerant legume crops, an investigation was conducted to compare the responses of Na^+^ and Cl^−^ separately with comparison to NaCl response in a soil-based system. High concentrations of both Na^+^ and Cl^−^ ions were found to have a negative effect on growth. Na^+^ was reported to interfere with K^+^ and Ca^2+^ nutrition disturbing stomata functions, which then affects photosynthesis. It is further known that Cl^−^ degrades chlorophyll, thus affecting photosynthesis [23]. In mungbean, little research has been performed on salt stress tolerance [26,27,28,29]. A set of microsatellite markers (SSRs) associated with salt tolerance was developed utilizing 12 mungbean genotypes [27]. There was also research on mycorrhizal fungi and growth hormones on mungbean growth under salinity. Rabbie G.H (2005) reported that arbuscular mycorrhizal fungi encourage growth of mungbean under salinity as compared to kinetic hormones [28]. When comparing mungbean to common bean, the salinity threshold levels of common beans are between the ranges of 30 to 100 mM NaCl [30,31,32,33]. A suitable mungbean salt test threshold was reported to be at 50 and 75 mM NaCl [34]. The reported threshold is an indication that both mungbean and common bean are both glycophyte legumes. However, studies to fully enumerate salt threshold levels and salt-tolerant *Vigna* spices are needed. 

In this study, the germination rate of a biodiverse mungbean germplasm set was tested under salt stress and was associated with genomic regions tagged with SNP markers. This is to identify SNP loci associated with salt-stress tolerance at the seedling stage.

## 2. Materials and Methods 

### 2.1. Plant Materials

Study materials consisted of a mungbean minicore collection of 297 accessions (Supplementary S1) [35]. The minicore panel represents the diversity of the mungbean collection held by the World Vegetable Center genebank [36]. The germplasm was split into 2 groups based on the agro-climatic conditions of the origins of the accessions. The germplasm of Group 1 was derived from arid/semiarid regions, comprising of accessions from India, Pakistan and the Middle East. Group 2 comprised accessions from tropic and subtropic regions of South East Asia, Oceania, Tropical Africa and Central/South America. A total of 284 genotypes were used, and genotypes with unknown origin were omitted. Seeds of these accessions were obtained from the World Vegetable Center gene bank, treated with a 1:5 diluted household bleach, aiming at a final concentration of 1% NaOCl solution for 3–4 h and then rinsed with distilled water.

### 2.2. Phenotyping

We used 50 mM NaCl to distinguish salt-sensitive accessions from salt-tolerant accessions in terms of seed germination. The 50 mM NaCl test benchmark was previously established using the same genotypes [37].

For testing the entire germplasm set, 10 seeds per accession for each of 2 replicates were used in the first trial. The seeds were placed in holes of polystyrene sheets floating in a 50 mM NaCl solution [37]. In a second experiment, 3 replicates of 10 seeds per accession were germinated on filter paper soaked with 50 mM NaCl in petri dishes. The experiments were arranged in a randomized complete block design. Temperatures ranged between 26 to 28 °C within a relative humidity of 56–66%, under 8/16 h of light/dark. Germination rate (in percent) was noted 5 days after sowing as follows:

[(Total number of seeds tested − Number of dead seeds)/Total number of seeds tested] × 100%.(1)

### 2.3. Test Statistics

Correlation of the phenotypic data from both the polystyrene sheets and petri dish regime was verified before using averages between 2 and 3 replications in downstream analyses. 

To investigate the degree of tolerance, 2 distinct germplasm groups representing accessions from (semi-) arid and from (sub-) tropical areas were created. A null hypothesis was set asserting no significant difference between the tolerance levels among the 2 groups. A t-test was conducted between the 2 groups to test the homogeneity of the variances between the two regional groups. We further tested for correlations between percentage germination, the agro-climatic origin of the accessions, and the population subgroups which we previously determined [35].

For GWAS, the phenotypic germination results were standardized by arc-log transformation using the SAS package in standardizing the outliers. Outliers were probed to avoid neither swelling nor suppression of the means. The standardized means were used for allele mining in GWAS.

### 2.4. Genotyping

A set of 5288 polymorphic SNP markers (minimum allele frequency at 0.05 and linkage disequilibrium r ≤ 0.1) generated by DartSeq [35] were utilized (Supplementary S2).

### 2.5. GWAS Models

Genome-wide SNP loci mining was performed using FarmCPU [38] and Genome Association and Prediction Integrated Tool (GAPIT)-mixed linear model (MLM) [39] enabled in the R environment to identify SNP loci associated with high seed germination rate under salt stress. 

The population structure of the mini core collection was determined [35] and used as a covariate in both FarmCPU and GAPIT-MLM to account for relatedness among genotypes. Associations were visualized by Manhattan and quantile-quantile (QQ) plots and associations above the significance threshold were identified. In correcting for multiple testing, Bonferroni threshold was set at 0.001. Minimum allele frequency was retained at 5%.

To uncover putative genes in associated genomic regions, the online J browser of the mungbean genome made available by the Crop Genomics Lab of Seoul National University (http://plantgenomics.snu.ac.kr/mediawiki-1.21.3/index.php/Main_Page) was utilized to search genes in the quantitative trait locus (QTL)-intervals.

## 3. Results

### 3.1. Variation of Salt Stress Tolerance During Germination

The overall performance under salt stress among the germplasm in the two difference testing regimes (hydroponic polystyrene sheets and petri dishes) revealed a uniform distribution (Table 1).

Results showed 5% of the germplasm accessions had high salt tolerance with more than 75% survival rate. We further noted that 13.13% (polystyrene sheet test) and 10.43% (petri dish test) of the genotypes in the two different testing regimes appeared to be in tolerant range (Table 1). Overall, the two test regimes showed correlations (r = 0.25, *n* = 297) at a high significant level (*p* < 10^−5^).

### 3.2. Salinity Tolerance among Geographical Origins

Grouping the minicore collection into two classes based on agro-climatic origins (i.e., Arid/Semi-Arid and Coastal Tropics/subtropics), the difference of the salinity tolerance level among the group was tested. T-tests revealed significant differences in salt tolerance between these groups (*p* < 0.001) (Table 2). Genotypes originating from the tropical/sub-tropics (38 genotypes) showed higher variance in salt-germination ability (118.40) as compared to 246 genotypes originating from arid/semiarid regions (82.92). On average, genotypes of (sub-) tropical origin had higher salinity tolerance than accessions from (semi-) arid environments. Furthermore, the panel showed a weak positive correlation (r = 0.228; *p*-value = 0.00011) between the origin of genotypes and their salt germinating abilities (Table 3). The high significance level of the correlation suggests that the geographical origin has an influence on the salt tolerance phenotype. 

### 3.3. GWAS Model

**Figure 1:**
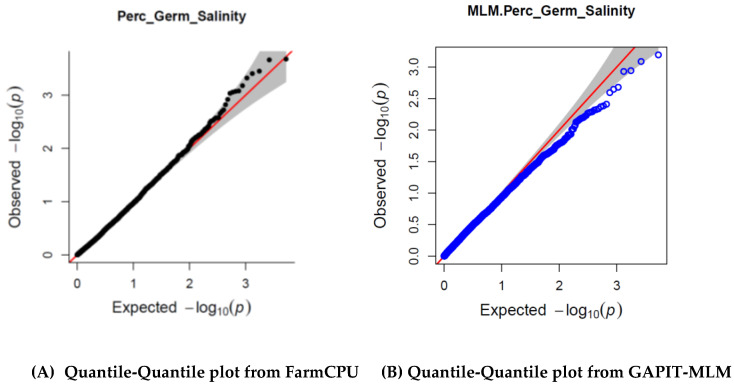
Quantile-Quantile (QQ) plots of FarmCPU (**A**) and GAPIT-MLM (**B**) genome-wide association study (GWAS) models.

Two GWAS models were tested—(1) FarmCPU, which uses a fixed linear model to give effect values of individual SNPs; and (2) GAPIT which uses the mixed linear model (MLM). For the SNP mining and analysis, the FarmCPU model was selected because q-q-plots suggested that there were some SNPs associated with salt tolerance. The alternative model GAPIT-MLM showed over adjustment that resulted in SNPs showing up beneath the chi-square null-hypothesis line (Figure 1).

This indicated that in this trait investigation, FarmCPU is a better model and was therefore utilized for SNP mining.

### 3.4. SNPs and Genes Associated with High Germination Rate under Salt Stress

**Figure 2:**
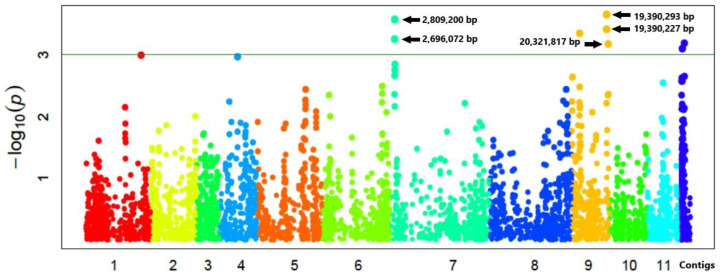
Manhattan plot of the association between single nucleotide polymorphisms (SNPs) and germination percentage of minicore collection accessions.

Association analysis identified two regions, on chromosome 7 and chromosome 9, respectively, to be significantly associated with salt stress tolerance at the seed germination stage (Figure 2; Table 4). The region on chromosome 7 stretched from position 2,696,072 to 2,809,200 bp, containing seven genes. Only gene, *Vradi07g01630* (Table 4), putatively encoding an ammonium transporter protein (AMT), was functionally annotated [40]. The other six genes within the region have no known functions that have been reported. The QTL on chromosome 9 stretched from 19,390,227 to 20,321,817 bp, containing 30 genes. Only two genes (*Vradi09g09510* and *Vradi09g09600*) have reported functions (Table 4). *Vradi09g09510* is homologous to the rice gene OsGrx_S16–encoding a glutaredoxin subgroup II protein; and *Vradi09g09600* encodes a dnaJ domain containing protein.

## 4. Discussion

### 4.1. Optimal Salt Concentration to Distinguish Salt Tolerant and Susceptible Accessions Based on Germination Rate

Ascertaining a NaCl test threshold is critical to investigate salt stress tolerance. The optimal salt test threshold varies among plant species, and in some cases, it varies within varieties of the same species [42]. For legumes, most salt test doses for tolerance testing ranged between 25–150 mM NaCl. Presently, there is no finite test benchmark in salinity studies of legumes. However, chickpea (25 to 60 mM NaCl) [43,44,45,46], soybean (50 to 150 mM NaCl) [47,48], common bean (30 to 100 mM NaCl) [30,31,32,33] and a basic mungbean salt test dose was previously reported at 50 and 75 mM NaCl [34]. We utilized 50 mM NaCl as our test dose threshold for mungbean salinity germination test according to Chung et al. (2016) [37]. 

### 4.2. Difference of Average Tolerance among Accessions Originating from Different Regions

Correlating genetic divergence and geographical diversity could only be ascertained when the origin of the genotypes can be accurately traced [49]. While genetic materials have been moved over time, it was reasonable to exclude genotypes with no defined origin and perform further assessments only with samples with secure information about their origin. Knowing that the genotyped minicore panel is heterozygous and diverse [16,17], it was assumed to be an ideal panel to identify alleles associated with high germination rate under salt stress. The results indicated that genotypes originating from tropical/subtropical areas displayed higher variability in their tolerance as compared to genotypes originating from arid/semiarid areas. The weak positive correlation (r = 0.228) with a significant *p* value (*p* value = 0.00011) between the origin of genotypes and their abilities to geminate under salt stress could be indicative that genetics does influence the trait under varying environments. 

### 4.3. Salt-Stress Loci and Putative Tolerance Genes

SNP markers showing an association (at Bonferroni-corrected −log10 (*p*) ≥ 3) were identified in chromosome 7 (two SNPs) and 9 (three SNPs). The region in chromosome 7 spanned approximately 113.128 kbp (from 2,696,072 to 2,809,200 bp). The associated region in chromosome 9 spanned 931.6 kbp (from 19,390,227 to 20,321,817 bp). The region in chromosome 7 contained one putative gene with a reported function, *Vradi07g01630*. In chromosome 9, two putative genes with reported functions were identified—*Vradi09g09510* and *Vradi09g09600*.

Gene *Vradi07g01630* in chromosome 7 is a homolog to an ammonium transporter gene (AMT) of Arabidopsis [40]. AMTs in plants can be divided into two subfamilies—AMT1 and AMT2 [50]. Physiological studies of ammonium uptake in roots revealed two systems, a high-affinity transport system (HATS), usually for low NH_4_^+^ concentration (in the micromolar range), and low-affinity transport system (LATS), for NH_4_^+^ concentrations in the millimolar range or higher [51,52,53]. AMTs play a vital role in sourcing and transporting ammonium in plants. Ammonium is known to be the vital source for plant nitrogen, obtained through plant cells via the AMTs in the plasma membrane and is then transported to the intracellular compartments such as the chloroplast, vacuoles and mitochondria [54]. In addition, leguminous plants take up ammonium generated via nitrogen fixation by rhizobia in root nodules, and there are specifically assigned AMTs as transporters from the bacteria to the cytoplasm [54].

There is little information about the relationship between ammonium transport, ammonium toxicity and salt stress tolerance [55]. Gene *PutAMT1;1* was identified and characterized in the wild perennial grass *Puccinellia tenuiflora*, a plant that is highly resistant to salts and high soil pH (>9.0) [56]. The study confirmed that *PutAMT1; 1* is an ammonium-inducible ammonium transporter. Overexpressing *PutAMT1; 1* in *A*. *thaliana* improved root to shoot growth, but increased the susceptibility to toxic methylammonium (MeA) by increasing the root to shoot mobility of MeA (or NH_4_^+^). The findings suggested that ammonium transport mitigates ammonia toxicity caused by salt stress. In that respect, our study can be corroborated that the mined region underlines a potential gene putative for salt stress.

The candidate gene on chromosome 9, *Vradi09g09510*, putatively encodes OsGrx_S16-glutaredoxin subgroup II protein [40]. OsGrx_S16-glutaredoxin subgroup II was found to be involved in cell wall organization or biogenesis. It is also responsible for the anatomical structure and morphogenesis of the plant. Furthermore, its extended function relates to tissue development. Glutaredoxins (GLXs) are antioxidants and are reported to be involved in stress responses [57]. However, while a few groups in the GLX family have had their functions identified, others still remained to be elucidated. The finding could corroborate the gene being significant in salt-stress-related functions.

The second QTL region in chromosome 9 contains the gene *Vradi09g09600*, annotated as dnaJ domain-containing protein [40]. DnaJ domains are present in stress-related proteins such as HSP40s (heat shock proteins), which are active in signaling pathways. HSP40 is a molecular chaperon very well-known for its functions in environmental stresses [58]. Apart from maintaining plant homeostasis during stress periods, it is responsible for many cellular processes such as protein folding, protein translocation across membranes, and regulation of protein degradation. Our results therefore tie down the region in chromosome 9 to be putatively associated to the dnaJ (HSP40) protein towards salinity stress in mungbean.

## 5. Conclusions

Mungbean is known to be a glycophyte, therefore 50 mM NaCl was used in the experimental test to distinguish between accessions that are tolerant and susceptible to salt stress during seed germination. There was a correlation between the germination rate under salt stress and the geographical origin of the genotypes. Genotypes from (sub-) tropical regions showed variation in germination under salt stress as compared to genotypes from (semi-) arid regions. This signifies trait variation amidst genotypes from different mungbean regions within the minicore panel.

Associated SNP loci were uncovered in both chromosome 7 and 9, elucidating putative gene homologs associated with salt stress related functions. Association of tolerance with multiple loci corroborated that salinity tolerance is a quantitative trait and multiple gene function does influence the trait expression. It could be attributed that the elucidated genes may possibly be associated with salt susceptibility. However, putative proteins mined within the associated gene regions proved to have stress-related functions.

It could also be assumed that rare variants do have contributions to the allelic effect of the trait. Genome-wide association analysis rather targets common variants. Allele frequency threshold and models to fix association power could possibly accommodate rare variants in GWAS mining. This could elucidate further insights into trait-controlled genes that were previously omitted by GWAS using common variants.

## Figures and Tables

**Table 1:** Salt tolerance phenotype distribution of the tested genotypes under hydroponics (polystyrene sheets) and in petri dishes.

Tolerance Level	Polystyrene Sheets	Petri Dish	Overall
Susceptible (0 to < 25%)	11	17	5
Moderately susceptible (25% to < 50%)	122	122	135
Moderately tolerant (50% to < 75%)	125	127	140
Tolerant (75% to 100%)	39	31	17

**Table 2:** T-test assuming unequal variances among the 2 agro-climatic origins.

Parameter	Arid Semi-Arid	Tropical Subtropical
Mean	45.413	51.838
Variance	82.922	118.403
Observations	246	38
Hypothesized Mean Difference	0.000	-
df	45	-
t Stat	−3.458	-
P(T ≤ t) two-tail	0.001	-
t Critical two-tail	2.014	-

**Table 3:** Correlations between percentage germination, regions and population subgroups.

Parameter	Germination %	Agro-Climatic Region	Subgroup
Germination %	1		
Agro-climatic region	0.228 **	1	
Subgroup	−0.117 **	−0.403	1

significant level at ** *p* < 0.01.

**Table 4:** SNP logarithm of the odds (LOD) scores, associated regions and gene homologs based according to the annotation given by Kang et al. (2014) [41].

Chr.	Position (bp)	Effect	LOD	Region Length (kbp)	Gene	Description Based on Arabidopsis
7	2,809,200	3.803	3.660	113.128	*Vradi07g01630*	ammonium transporter protein (AMT)
7	2,696,072	−3.629	3.323			
9	19,390,293	−3.069	3.675	931.590	*Vradi09g09510*	OsGrx_S16-glutaredoxin subgroup II
9	19,390,227	2.892	3.450		*Vradi09g09600*	dnaJ domain protein (HSP40s-heat shock protein)
9	20,321,817	2.963	3.178			

## Supplementary Materials

The following are available online at https://www.mdpi.com/2073-4425/11/7/759/s1, Supplementary S1: mungbean accession and trait data; Supplementary S2: SNP markers.
